# The Study of the Effect of Blade Sharpening Conditions on the Lifetime of Planar Knives During Industrial Flatfish Skinning Operations

**DOI:** 10.3390/ma18133191

**Published:** 2025-07-06

**Authors:** Paweł Sutowski, Bartosz Zieliński, Krzysztof Nadolny

**Affiliations:** 1 Faculty of Mechanical and Energy Engineering, Department of Production Engineering, Koszalin University of Technology, Raclawicka 15-17, 75-620 Koszalin, Poland; pawel.sutowski@tu.koszalin.pl (P.S.); bartosz.zielinski@espersen.com (B.Z.); 2 Espersen Poland Ltd., BoWiD 15 Str., 75-671 Koszalin, Poland

**Keywords:** technical knives, grinding conditions, lifetime, multi-objective optimization, cooling conditions

## Abstract

Users of technical blades expect new generations of tools to feature reduced power requirements for process and maximized tool life. The second aspect is reflected in the reduction in costs associated with the purchase of tools and in the reduction in process line downtime due to tool replacement. Meeting these demands is particularly challenging in cutting operations involving heterogeneous materials, especially when the processed raw material contains inclusions and impurities significantly harder than the material itself. This situation occurs, among others, during flatfish skinning operations analyzed in this paper, a common process in the fish processing industry. These fish, due to their natural living environment and behavior, contain a significant proportion of hard inclusions and impurities (shell fragments, sand grains) embedded in their skin. Contact between the tool and hard inclusions causes deformation, wrapping, crushing, and even chipping of the cutting edge of planar knives, resulting in non-uniform blade wear, which manifests as areas of uncut skin on the fish fillet. This necessitates frequent tool changes, resulting in higher tooling costs and longer operating times. This study provides a unique opportunity to review the results of in-service pre-implementation tests of planar knives in the skinning operation conducted under industrial conditions. The main objective was to verify positive laboratory research results regarding the extension of technical blade tool life through optimization of sharpening conditions during grinding. Durability test results are presented for the skinning process of fillets from plaice (*Pleuronectes platessa*) and flounder (*Platichthys flesus*). The study also examined the effect of varying cooling and lubrication conditions in the grinding zone on the tool life of technical planar blades. Sharpening knives under flood cooling conditions and using the hybrid method (combining minimum quantity lubrication and cold compressed air) increased their service life in the plaice skinning process (*Pleuronectes platessa*) by 12.39% and 8.85%, respectively. The increase in effective working time of knives during flounder (*Platichthys flesus*) skinning was even greater, reaching 17.7% and 16.3% for the flood cooling and hybrid methods, respectively.

## 1. Introduction

Technical knives are a category of specialized cutting tools widely used across various manufacturing sectors. For example, they can be used for cutting paper into sheets [1,2], cutting textiles [3], cutting sheet metal [4], or plastics [5]. A significant portion of technical knives’ applications occurs within the food processing industry. In this context, blades are utilized for separating parts of plants or animals, shredding, and operations such as fish skinning [6,7].

Fish processing challenges are the focus of research by numerous companies and scientific institutions worldwide. Several relevant studies and technological improvements have been reported by researchers such as Viatcheslavovich, Ageev, Chu, and Komlatsky.

Viatcheslavovich et al. developed mathematical models describing the resistance forces acting on knives without side edges, showing that these forces depend significantly on the geometry of the knife (sharpening angle, thickness), cutting speed, and the rheological properties of the material [8]. This foundational research provides essential parameters that can be used to model cutting processes in fish processing. Similarly, Ageev et al. focused on optimizing the sharpening angle to minimize cutting resistance, deriving mathematical models that incorporate normal contact pressures and other key mechanical parameters [9,10]. These findings directly inform the design and cutting efficiency of the tools, which are crucial to the precision of fish processing lines. Chu et al. explored the application of advanced blade coatings, such as Zr- and Fe-based thin-film metallic glasses and Teflon, to reduce cutting forces [11]. Their findings suggest material innovations that could prolong blade life and enhance performance, contributing to cost-effective and efficient fish processing systems. Komlatsky et al., on the other hand, addressed the broader context of automation in the fish industry, describing the architecture and functionality of systems for receiving, freezing, sorting, cutting, and other tasks related to the processing and storage of fish and fish products [12]. Their work underscores the importance of integrating optimized cutting tools and methods into comprehensive automated solutions. An extensive review of fish cutting techniques—including deheading, filleting, portioning, skinning, and trimming—was provided in [7]. This article specifically concentrates on assessing the quality of skinning operations performed by 14 out of the 24 companies reviewed in 2022 [7].

Together, all of these studies can point to methodological frameworks and technical choices in subsequent research, particularly in the areas of sharpening methods and the impact on the service life of technical knives in actual skinning applications, as well as the integration of these components into automated fish processing systems.

Manufacturers using technical knives demand tools that minimize the required cutting forces. This, in turn, reduces the power consumption of processing equipment. In addition, they require tools that exhibit the longest possible service life. A longer tool life contributes to lowering costs associated with the purchase of new tools and reduces process line downtime resulting from tool replacement. Meeting the last postulate is particularly difficult in the case of cutting operations of heterogeneous materials (of varying hardness) and when the processed raw material may have inclusions or impurities of much greater hardness than the primary material. This scenario is typical in flatfish skinning within the fish processing industry. Due to their natural behavior, flatfish tend to have a higher concentration of hard inclusions and impurities embedded in their skin—such as shells and sand grains—compared to other fish species, as demonstrated in [13].

While the flat shape of the fish enables relatively straightforward mechanization and automation of the skinning process, the presence of hard inclusions in the skin significantly reduces the lifespan of the cutting tools. The challenges of automating fish processing are being addressed not only by machinery manufacturers (such as NOCK Maschinenbau GmbH, Germany, and Cretel by ATS, Belgium) but also by research centers. Arnþórsdóttir et al. presented results from fish processing studies (filleting, trimming, and skinning) conducted under industrial conditions at Festi ehf., a fish processing plant in Iceland [14]. The authors used the Baader machines (Baader, Lübeck, Germany)—the fish were deheaded in a Baader 413, filleted with a Baader 189, and skinned with a Baader 51 machine. Recent publications cover topics such as robotic fish processing lines enhanced by machine learning [15], and computer vision and deep learning techniques for identifying gaping in salmon fillets [16].

Industrial practice shows that contact between the tool and hard inclusions causes plastic deformation, wrapping, crushing, and even chipping of the cutting edge of planar knives. This leads to uneven blade wear, manifested by defects during cutting, such as unremoved skin on the fish fillet. Accelerated tool wear in the skinning operation—integrated within an automated production line—results in frequent line stoppages for knife replacement. This not only causes undesirable increases in tooling costs but also limits the efficiency and throughput of the production system, which operates at a forced (synchronized) pace.

The above-mentioned challenges motivate scientific research aimed at finding solutions to extend the lifetime of such tools. Possible approaches include applying protective coatings to cutting tools using PVD-based processes or locally hardening the blade structure through methods such as shot peening or laser shock peening technologies.

The first solution, widely used in woodworking technology, can lead to contamination of the product with abrasive particles or peeling of the protective coating. The issue of applying protective layers to extend the life of technical blades is discussed, for example, in [17,18,19]. The second solution, on the other hand, is applied to reduce surface roughness, enhance hardness, and increase the densification of the surface layer’s microstructure, thereby contributing to the strengthening of the material. These topics are extensively reviewed in [20]. Nonetheless, it may be assumed that during the processing of fish fillets, apart from the anticipated improvement in mechanical strength, the hardening of the blade structure could contribute to an increased propensity for chipping, as the material becomes more brittle as a result of the hardening process. Consequently, in this case as well, there is an increased risk of contamination of the processed raw material by fragments originating from the blade material.

An increase in the lifetime of knives can also be achieved by optimizing the blade geometry and sharpening conditions during the grinding process. The grinding process of industrial planar (rectilinear) blades is carried out by inducing a relative rotary motion of the abrasive tool along the edge of the blade. The grinding wheel, however, can contact the machined surface with its periphery (peripheral grinding), its face (face grinding), or a specially shaped conical surface (conical grinding). In each of these variants, the final trajectory of abrasive grain movement is determined by the feed rate, machining allowance (both for the roughing and sparking-out phases), and the kinematics of the grinding process. Research conducted in this field has shown a statistically significant correlation with knife sharpness, expressed in terms of cutting force measured during fish processing tests. This issue was further analyzed by the authors and discussed in detail in [21].

The factors identified as negatively affecting the service life of technical blades used in the processing of flatfish led the authors of this paper to develop a new technology for shaping the geometry of blades during the grinding process. Extensive experimental research on this process, described in [13,22,23,24,25,26], allowed for the selection of its most advantageous parameters as well as the blade geometry in terms of the cutting force obtained under laboratory conditions. The aim of the experiments described in this article was to compare the knowledge gained in the applied research stage conducted on experimental stands with the results of pre-implementation research carried out under industrial conditions. Such tests consider all possible disruptions resulting from the mass production regime of food products, which cannot be reproduced in the laboratory. This makes it possible to determine the actual effect of the modifications introduced and to identify any factors limiting their scope of application.

Technological parameters for the regeneration (grinding) of planar knives were selected, and their service life was verified during the skinning of European plaice (*Pleuronectes platessa*) and European flounder (*Platichthys flesus*) fillets. The study also investigated the effect of different cooling and lubrication conditions in the grinding zone on the service life of industrial planar blades. The research was conducted at the medium-sized fish processing plant Espersen Poland Ltd. (Koszalin, Poland), and the results obtained using brand-new tools—typically employed in the described process—served as a reference. This provided a unique opportunity to assess the actual impact of grinding process conditions on the service life of knives operating under typical industrial conditions, characterized by disturbances that are difficult or impossible to replicate in laboratory settings.

The paper is structured as follows: Section 2 provides a detailed description of the materials and methods used in the research, including the procedure for selecting optimal grinding process conditions (Section 2.3) and details of the knives’ operational experiments (Section 2.4); Section 3 discusses the experimental results related to tool life; and Section 4 presents the conclusions.

## 2. Materials and Methods

The operational testing of technical knives used in the skinning process of flatfish was conducted under industrial conditions at the processing facility of Espersen Poland Ltd. (Koszalin, Poland). The primary objective of the study was to determine the service life of planar knives whose blade geometry had been shaped under the most favorable grinding conditions, as identified through prior experimental research. The findings of these experimental investigations have been comprehensively detailed in the publication [21]. This approach enabled the verification, under actual industrial operating conditions, of the performance characteristics of technical blades formed under the specifically selected grinding parameters.

As part of the experimental investigations, particular attention was directed toward evaluating the impact of different cooling and lubrication strategies applied in the grinding zone on blade quality, understood primarily in terms of tool life. The grinding operations were performed using three distinct coolant application methods: conventional flood cooling (WET), minimum quantity lubrication (MQL), and a hybrid strategy (HYB) incorporating compressed air assistance (CAG, using cold air guns). The main objective of the comparative analysis was to determine the extent to which the selected cooling method influences the repeatability of edge shaping, the condition of the surface layer, and the actual tool life under industrial conditions. This approach enabled a comprehensive assessment of the effectiveness of each cooling-lubrication strategy from both operational and environmental perspectives and provided a foundation for developing practical recommendations regarding the selection of cooling techniques in the refurbishment process of technical knives.

### 2.1. Materials—Characterization of Soft Tissue Specimens

The raw material used in the skinning trials consisted of fillets from *Pleuronectes platessa* (European plaice) and *Platichthys flesus* (European flounder), sourced from the Baltic Sea fishing grounds (FAO Area 27 IIId/25; Port of Kołobrzeg, Poland). The fishing method was identified as Otter Trawl Bottom (OTB). During processing, the temperature of the raw material was maintained consistently within the range of 0–3 °C for both species. Additional characteristics of the processed fish tissue are provided in Table 1.

**Figure 1:**
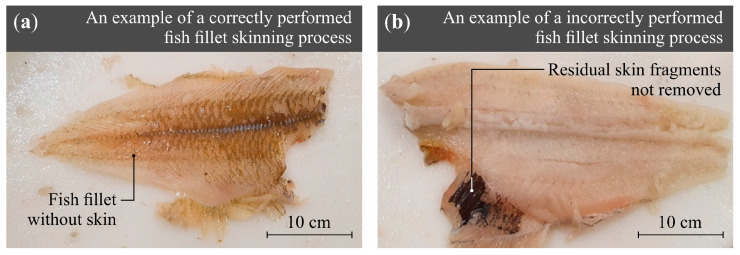
View of a *Pleuronectes platessa* fish fillet after the skinning process: (**a**) following a properly executed procedure; (**b**) exhibiting typical defects of the process (residual skin fragments not removed).

The production process at Espersen Poland Ltd. is carried out up to the point at which the allowable threshold of residual skin on processed fish fillet portions is exceeded. Internal quality control standards permit a maximum of 7% residual skin remaining on the fillet surface, relative to the total skin area intended for removal. A representative image of a fish fillet following a correctly performed skinning operation is presented in Figure 1.

**Figure 2:**
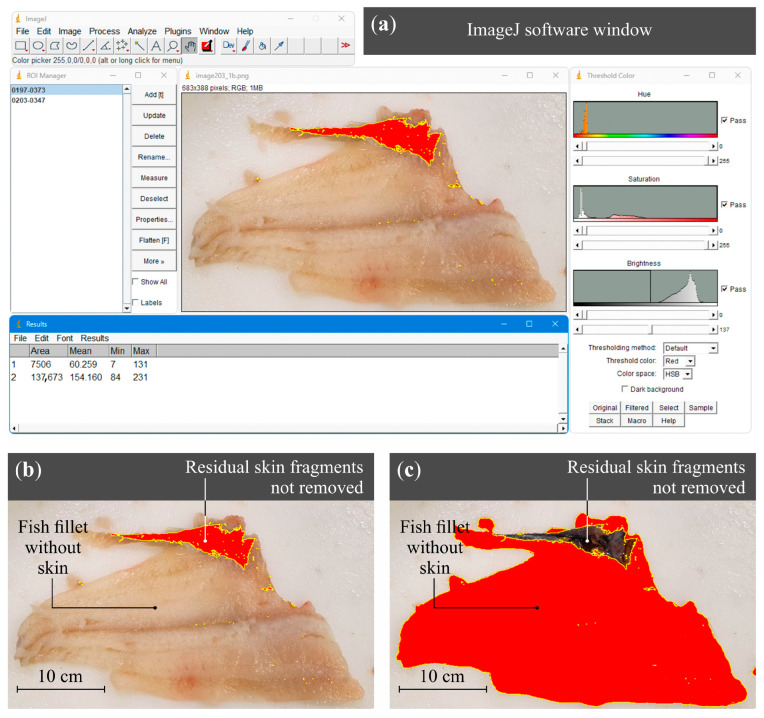
Determination of the surface area affected by characteristic skinning defects: (**a**) thresholding and quantitative analysis of the region of interest; (**b**) marked example of an area with residual skin fragments; (**c**) marked example of an area where the skin was properly removed.

On the production line, inspection is conducted using vision systems (industrial inspection cameras) that detect differences in color and texture between flesh and skin. Fillets with excessive skin residues are automatically rejected. In the conducted study, defect identification was performed through visual assessment followed by optical analysis of images using ImageJ software (developed by Wayne Rasband, ver. 1.54 m). An example of the analysis of the area ratio occupied by residual skin on the processed fillet relative to the total skin area intended for removal is shown in Figure 2. The area occupied by residual skin was quantified through image processing using the color threshold function. This method involves defining a specific range of pixel color values that correspond to the residual skin, enabling its segmentation from the fillet tissue in digital images.

Thresholding in the HSB color space was applied to segment residual fish skin from the surrounding tissue. The threshold settings used were as follows: Hue: 0–255, Saturation: 0–255, and Brightness: 0–137. This configuration allowed for the selection of all color tones and saturation levels while focusing exclusively on the darker regions of the image. The rationale behind this approach was based on the visual characteristics of residual skin, which typically appears darker than both the underlying fish muscle and the white cutting surface.

The choice of the HSB color space, and specifically the restriction in the brightness channel, is consistent with established methods for isolating features with low luminance contrast in biological image analysis. This approach enables robust segmentation in cases where the color hue and saturation may vary due to lighting or specimen variability, but where brightness remains a consistent discriminating factor. An increasing number of studies have confirmed the effectiveness of the HSB color space in accurate skin segmentation, particularly in the context of biological material analysis. Notable examples include articles [27,28,29].

This methodology and threshold ranges were established in consultation with the Quality Manager at the processing facility where the experiments were conducted, to ensure that the detection criteria aligned with practical standards for identifying residual skin fragments.

Following the thresholding procedure, the residual skin area was measured as 7506 pixels, while the remaining fillet area was 137,673 pixels. Consequently, the ratio of residual skin to the total skin area targeted for removal was calculated as 7506 to 145,179 pixels (5.17%). This quantitative approach allowed a precise assessment of the skinning process efficacy by objectively measuring skin remnants on processed fillets.

### 2.2. Materials—Planar Knives

Both groups of flat industrial knives intended for fish skinning in this study—factory-new blades and those formed through a controlled grinding process—were manufactured from martensitic stainless steel X39Cr13 (1.4031) by Kuno Wasser GmbH (Solingen, Germany) for Steen F.P.M. International (Kalmthout, Belgium). The material contained approximately 13.5% chromium, and its full chemical composition is presented in Table 2.

According to standard BS EN 10088-3:2023 [30] and BS EN 10088-1:2014 [31], the mechanical and physical properties of X39Cr13 steel (at room temperature) are summarized in Table 3. A Brinell hardness of up to 245, providing the cutting edge with good wear resistance during fish skinning, thereby extending the tool’s service life. The yield strength of 650 MPa ensures that the blade maintains its shape and withstands mechanical loads without permanent deformation, which is crucial for maintaining cutting precision. Tensile strength (*R_m_*) ranging from 800 to 1000 MPa indicates high resistance of the material to cracking and mechanical damage, contributing to operational safety and reliability. A minimum elongation at fracture of 10% reflects sufficient ductility, enabling absorption of impact forces and reducing the risk of blade fracture when encountering hard elements. Additionally, an impact energy (ISO-V, longitudinal) of *KV* ≥ 12 J provides resistance to sudden shocks and impacts, which is essential under demanding working conditions and enhances the durability of the knives during prolonged use.

### 2.3. Selected Conditions for the Grinding Process

The investigation concerning the identification of appropriate sharpening parameters for planar knives was conducted and is comprehensively described in [21]. Based on the obtained results and the developed mathematical models, it can be concluded that the magnitude of the spark-out feed, the feed rate, and the kinematics of the grinding process exert a significant influence on the grinding power. Consequently, these factors directly affect the cutting force generated during the operational use of the knives.

The study also demonstrates that the most effective grinding kinematics in this context is peripheral grinding using a grinding wheel without a conical edge. Accordingly, the models describing the grinding power (*P*) and cutting force (*F_c_*) can be expressed by the following equations:

(1) $$P=-14.274\ln\left(GT\right)-11.542\ln\left(v_{f}\right)+28.389\ln\left(a_{es}\right)+11.412\ln\left({GT}_{val}a_{es}\right)+33.287\ln\left(v_{f}a_{es}\right)+209.94,$$

(2) $$F_{c}=11.8476+27423068.498\exp\left(-1403.051a_{es}+0.43736v_{f}a_{es}+23146.7544a_{es}^{2}\right),$$

where: *GT* is a categorical variable representing the type of grinding, while *GT_val_* denotes the numerical value assigned to the category (for peripheral grinding, *GT_val_* = 5.157), *v_f_* is feed speed, and parameter *a_es_* is the allowance for sparking-out phase used in the grinding process.

For peripheral grinding, the power model can be written in a simplified form:

(3) $$P=205.2453-11.542\ln\left(v_{f}\right)+39.801\ln\left(a_{es}\right)+33.287\ln\left(v_{f}a_{es}\right).$$

The models presented in Equations (2) and (3), as well as their respective explanatory variables, exhibit statistical significance, as demonstrated in our previous study [21]. The level of fit of the model to the data (*R*^2^_adj._) is equal to 0.862 and 0.843 for power and cutting force, respectively.

**Figure 3:**
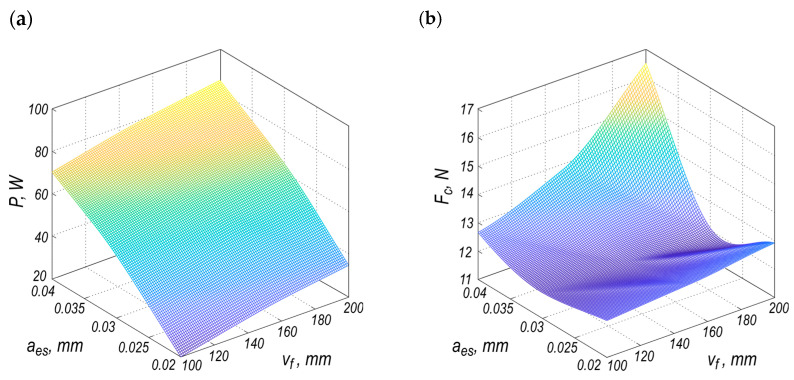
Peripheral grinding models as a function of the machining allowance (*a_es_*) and the feed speed (*v_f_*) for: (**a**) grinding power (*P*); (**b**) cutting force (*F_c_*) [21].

The plots of these models are presented in Figure 3. Both surfaces, which represent the range (image) of the functions in three-dimensional space over the domain defined by the independent variables, exhibit distinctly different characteristics. They differ in terms of geometric shape, which is manifested in the divergent structure of extrema (critical points such as maxima and minima), as well as in the nature of the variation in function values across the space of independent variables.

As shown in Figure 3a, grinding power (*P*) increases nearly linearly with both feed rate and machining allowance. However, the feed rate (*v_f_*) has a noticeably greater effect on power consumption, due to the steeper gradient along the *v_f_* axis. This suggests that increasing the feed rate results in a significantly higher power demand, probably due to the increased material removal rate during grinding.

Figure 3b shows that the cutting force (*F_c_*) has a more complex, nonlinear relationship. Both parameters, *a_es_* and *v_f_*, affect the cutting force. Therefore, it is difficult to identify the dominant factor affecting mechanical resistance during the cutting operation. At higher feed speeds, the machining allowance (*a_es_*) appears to have a stronger effect than the feed rate.

Overall, the data suggest that feed speed is the main factor affecting energy consumption, while the cutting force value is influenced by a combination of the two parameters. These insights are important for optimizing process parameters to balance energy efficiency and tool wear.

**Figure 4:**
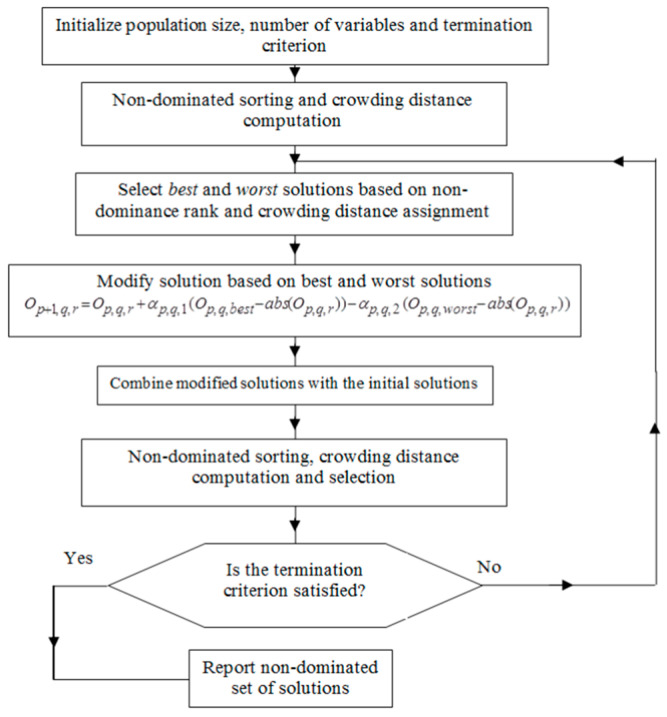
Flowchart of the MO-Jaya algorithm by R. Venkata Rao [32].

In order to unambiguously determine the grinding parameters for knives that can be considered optimal, a multi-objective optimization (MOO) approach was employed. For this purpose, the multi-objective Jaya (MO-Jaya) algorithm was utilized. This algorithm has been applied in various manufacturing processes, including plasma arc machining, electro-discharge machining, and micro-electro-discharge machining [32]. The Jaya algorithm was proposed in 2016 by R. Venkata Rao as a method that is simple to implement, as the candidate solution is updated in a single phase using only one equation [33]. To solve multi-objective optimization problems (MOOP), the algorithm illustrated in Figure 4 was adopted.

This algorithm ensures the generation of a set of Pareto-efficient solutions for a multi-objective optimization problem (MOOP). To avoid the clustering of solutions around a single point, all high-quality solutions within the search space are identified based on the value of ξ (crowding distance). A solution with a higher rank and a higher ξ value is considered the best candidate for the next generation (iteration). As a result, a solution originating from a more isolated region of the search space is preferred over one located in a densely populated region of the search space [32]. Each individual crowding distance (*ξ_j_*) is an estimate of the density of the solutions as follows:

(4) $$\xi_{j}=\xi_{j}\frac{f_{m}^{j+1}-f_{m}^{j-1}}{f_{m}^{max}-f_{m}^{min}},$$

where *f_m_* is the objective function value of the *m*-th objective of the *j*-th solution—the highest and the lowest values of the *m*-th objective function in the population.

In order to identify a single optimal solution from the set of Pareto-optimal solutions, the distance minimization technique relative to the ideal point was employed as the selection strategy. Within the solution search space, the ideal point can be defined as follows:

(5) $$P_{\left(ideal\right)}=19.46,$$

(6) $$F_{c\;(ideal)}=11.91,$$

These correspond to grinding power and cutting force, respectively, where *P*_(*ideal*)_ denotes the minimum attainable grinding power, and *F*_*c* (*ideal*)_ represents the minimum attainable cutting force that has been qualified for inclusion in the Pareto front.

The set of Pareto-efficient solutions provided by the MO-Jaya algorithm is reported in Table 4.

For each point in the set of Pareto-optimal solutions, the distance to the ideal point was calculated using the Frobenius norm [34]:

(7) $${\left|\left|A\right|\right|}_{F(i)}=\sqrt{{\left(P_{i}-P_{min}\right)}^{2}+{\left(F_{ci}-F_{cmin}\right)}^{2}}.$$

The best single solution corresponds to: *P* = 19.46 W, *F_c_* = 12.30 N for the independent variables: *v_f_* = 100 mm/min and *a_es_* = 0.02 mm. The final selected grinding process parameters for planar knife edge shaping, intended for experimental and industrial operational studies, are presented in Table 5.

The grinding process constitutes one of the key finishing operations in the machining of engineering materials. It is characterized by high dimensional accuracy and the quality of the resulting surface finish. Simultaneously, it is a process that generates a significant amount of heat in the contact zone between the grinding wheel and the workpiece. Consequently, cooling and lubrication of the grinding zone are applied using appropriate coolants, such as water-based oil solutions.

**Figure 5:**
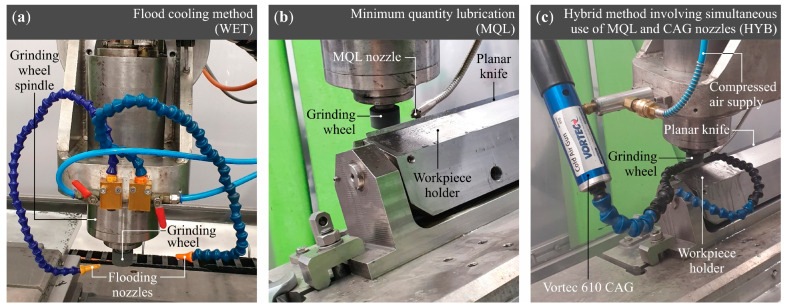
View of the grinding zone configuration enabling the application of: (**a**) flood cooling method (WET), (**b**) minimum quantity lubrication (MQL), and (**c**) hybrid method involving simultaneous use of MQL and CAG nozzles (HYB).

A conventional cooling technique is the flood cooling method (WET grinding), which involves the intensive supply of a large volume of coolant–lubricant fluid to the machining zone. The drawback of this method is the use of substantial quantities of process fluids, leading to high operational costs, the necessity of employing advanced filtration and disposal systems, as well as potential risks to operator health and the environment. In light of the above, the experimental grinding process research program was supplemented by the introduction of an additional variable factor, namely the method of cooling and lubrication of the grinding zone. Grinding was performed under conditions of conventional flood cooling (WET), minimum quantity lubrication (MQL), and the supply of cooled compressed air through a nozzle (CAG), as illustrated in Figure 5. These tests aimed to determine the most advantageous conditions for delivering cooling and lubricating agents to the machining zone.

Although partial quality criteria of the process, such as the amount of consumed coolant, presence of defects, microstructural changes of the surface after grinding (e.g., surface burns, residual stresses, microcracks, as well as deterioration of the fatigue properties of the component), or grinding wheel wear, are important from the perspective of machining process analysis, they do not provide an unambiguous answer as to whether the tool will perform its function optimally in practice. Therefore, the operational performance of the knife—understood as its tool life, i.e., the ability to maintain sharpness, wear resistance, and stability of cutting parameters during service—was adopted as an integrated criterion, comprehensively accounting for the effects of all significant phenomena occurring during grinding.

The adopted grinding process conditions and the designations of the planar knives involved in the operational tests are provided in Table 6. Detailed information on the initial condition (after sharpening in the grinding process) of the knives tested can be found in [24].

### 2.4. Details of Operational Experiments on Knives

**Figure 6:**
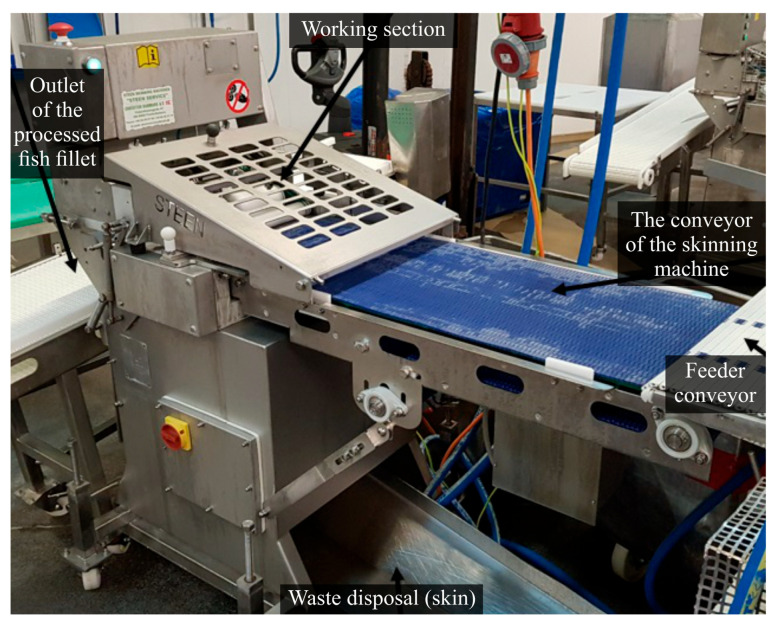
General view of the Steen ST 600 skinning machine on the production line at the Espersen Poland Ltd. manufacturing facility.

Operational tests of knives selected based on the results of preliminary studies were conducted under production conditions at Espersen Poland Ltd. using a Steen Skinning ST 600 skinner machine (F.P.M. INTERNATIONAL NV, Belgium), which is an integral part of the technological processing line for flatfish, as shown in Figure 6.

Prior to deployment in the food processing facility, the knives underwent a procedure aimed at achieving the required level of cleanliness and sterility to enable safe contact with food products. The procedure comprised the following steps:

- washing in a solution prepared by mixing 100 mL of the chemical agent Diversey Sureclean Plus VK9 per 10 L of water (Diversey Inc., Fort Mill, SC, USA);
- rinsing under running water;
- sterilization at a temperature of 82–85 °C for 10 min;
- application of a sprayed alcohol-based chemical agent Diversey Divodes FG VT29 (Diversey Inc., Fort Mill, SC, USA), which is a ready-to-use solution that evaporates shortly after application, leaving the knives in a condition suitable for use in food processing.

The skinning process was conducted using both new, commercially available reference knives and knives shaped under grinding process conditions resulting from prior studies. Experiments were performed on the same batch of raw material in order to eliminate confounding factors that could affect the validity of the comparative results.

**Figure 7:**
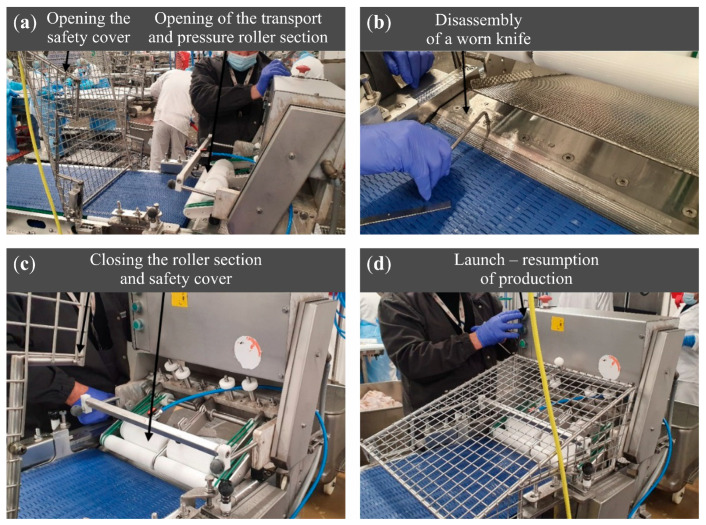
Selected stages of the Steen Skinning ST 600 machine changeover: (**a**) opening of the cover and transport and pressing roller section; (**b**) removal of the worn blade and installation of a new one; (**c**) closing of the working section and safety cover; (**d**) machine start-up.

Due to the requirement to replace the knives during successive production cycles in the operational tests, the preparation procedure of the skinning machine for operation was performed each time (Figure 7):

- the safety cover of the working section was raised, and the pressing rollers and transport rollers section was opened;
- the working parts of the skinning machine were rinsed with running water to remove residues of the processed raw material;
- the clamping screws of the knife support were unscrewed, and the worn knife was removed, followed by installation of a new reference knife or a knife regenerated through the grinding process;
- after tightening the clamping screws of the knife support, the pressing and transport rollers section along with the safety cover was closed;
- production was resumed, and timing measurements were initiated.

The production process was continued until the allowable threshold of skin residue on the processed fish fillet portions was exceeded. Internal plant standards permit a maximum of 7% skin residue on the processed fillet relative to the total removed skin area. The termination of the production cycle was equivalent to stopping the time measurement.

To determine the weight of processed raw material per unit of time, containers for collecting fish portions after the skinning process were used. The mass of the processed fish was measured using a platform scale with a measurement accuracy of ±100 g.

The described procedures were carried out in the same manner for three reference (new) knives and nine knives shaped under grinding process conditions determined based on the results of preliminary studies. The edge shaping parameters of the factory-new knives (Kuno Wasser GmbH) are proprietary, whereas the grinding parameters of the knives selected in previous studies (Koszalin University of Technology) are provided in the preceding chapter, in Table 5.

Table 7 presents a list of parameters and environmental conditions applied during the processing (skinning) of fish raw material. Table 8 provides the designations of planar knives selected for operational testing in the soft tissue cutting (skinning) process, along with the corresponding edge shaping conditions applied during grinding. The knives were divided into two main groups (Reference group and Ground knife group) and further categorized according to the cooling method employed during grinding (MQL, CAG, and hybrid). Knives shaped under cooling conditions using chilled compressed air (via CAG nozzle) were excluded from the operational tests due to unfavorable quality characteristics identified in preliminary studies. The number of repetitions for individual plan points was set at three, resulting in a test program involving 12 knives, applied both in flounder skinning and smooth skin fish skinning tests.

## 3. Results and Discussion

The results obtained from the plaice (*Pleuronectes platessa*) skinning process are compiled in Table A1 (Appendix A). The findings indicate variable tool life durations until loss of cutting ability, and consequently, different quantities of processed raw material depending on the knife group analyzed. The tool life of the reference knives was determined to be on average 113 min, enabling the processing of a total of 474 kg of plaice over 5.65 working hours (339 min). Experiments conducted for the remaining knife groups showed comparable or improved performance. The average tool life across all groups was 118.5 min, corresponding to the processing of approximately 166 kg of raw material.

**Figure 8:**
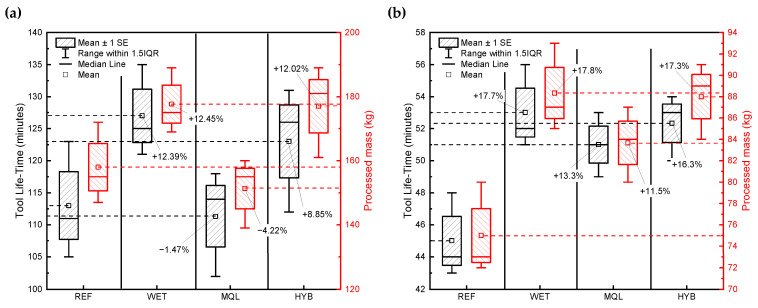
Comparison of effective tool life and processed material mass for planar knives used in industrial skinning under four cooling strategies (REF—reference knives, WET—conventional flood cooling, MQL—minimum quantity lubrication, HYB—hybrid cooling), for: (**a**) plaice (*Pleuronectes platessa*), (**b**) flounder (*Platichthys flesus*).

Figure 8 presents a comparative analysis of effective tool life and processed material mass for planar knives used in the industrial skinning of two fish species—(a) plaice (*Pleuronectes platessa*) and (b) flounder (*Platichthys flesus*)—under four different cooling strategies: reference (REF), conventional flood cooling (WET), minimum quantity lubrication (MQL), and hybrid cooling (HYB).

The primary vertical axis (left, in minutes) represents the effective tool life (time until performance degradation), while the secondary vertical axis (right, in kilograms) shows the total mass of material processed before tool replacement. For each cooling strategy, box plots summarize the distribution of measured tool life, with the median, mean (marked with a square), and interquartile ranges presented. Red bars represent the processed mass, aligned with the red axis on the right.

Each boxplot represents the distribution of the collected data. The elements of the boxplots are as follows:

- the central horizontal line inside each box denotes the median;
- the top and bottom edges of the box represent the upper and lower quartiles, respectively—together defining the interquartile range (IQR);
- whiskers extend to the minimum and maximum values within 1.5× IQR from the quartiles, showing the spread of the central portion of the data;
- hatched bars (mean ± SE) represent the mean tool life along with the standard error (SE) of the mean—indicating the precision of the mean estimate.

The results presented in both figures clearly demonstrate the varying effectiveness of the applied cooling strategies in terms of tool life and the mass of processed material. For plaice (Figure 8a), the reference strategy (REF) results in a median tool life slightly below 112 min and a processed mass of approximately 155 kg. In the case of flounder (Figure 8b), the same general trends are observed, though the absolute values are lower due to species-specific characteristics. The REF strategy results in a median tool life of around 44 min and a processed mass close to 73 kg. Other observed results indicate that, under the adopted grinding conditions, the cutting properties of the knife edges were successfully restored. As a result, the knives could be reused in the skinning process without exerting any significant influence—either beneficial or detrimental—on tool life.

### 3.1. Statistical Analysis of Significance

Beginning with the analysis of results for the flounder (*Platichthys flesus*), the first output factor, the operating time of the knife in the skinning process, was subjected to a general ANOVA and Fisher’s test (pairwise mean comparison). General ANOVA analysis (Table 9) revealed a test statistic *F*-value of 7.16 and a *Prob* > *F* of 0.012, indicating statistically significant differences between the means of the groups at a significance level of *α* = 0.05. This confirms a significant influence of cooling conditions in the grinding process on the operating time of the knife in skinning.

**Figure 9:**
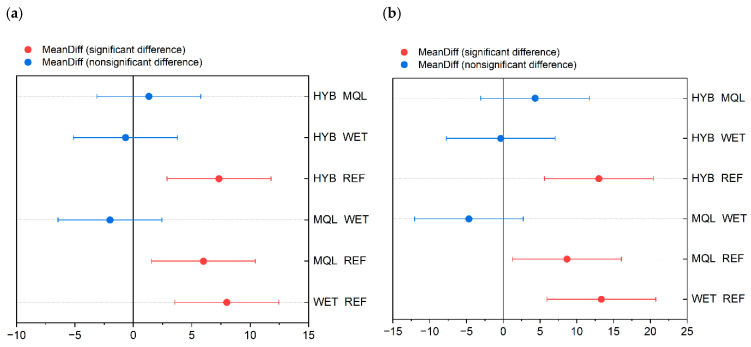
Means comparison plot for four different cooling strategies: reference (REF), conventional flood cooling (WET), minimum quantity lubrication (MQL), and hybrid cooling (HYB), with respect to: (**a**) the lifetime of technical knives, and (**b**) the mass of processed flounder fish (*Platichthys flesus*).

Fisher’s test (Table A4, Appendix A) further identified statistically significant differences (*Sig* = 1) at α = 0.05 for the following pairwise comparisons: WET vs. REF (with mean difference between data groups *MeanDiff* = 8, and probability of observing a test statistic *Prob* = 0.003), MQL vs. REF (*MeanDiff* = 6, *Prob* = 0.014), and HYB vs. REF (*MeanDiff* = 7.33, *Prob* = 0.005). These results clearly demonstrate that all examined cooling conditions (WET, MQL, and HYB) differ significantly from the reference condition in terms of their effect on knife operating time. The Fisher LSD plot (Figure 9a) visually confirms these findings.

Proceeding to the second output factor for flounder (*Platichthys flesus*), the mass of processed raw material in the skinning process, the general ANOVA (Table 9) revealed an *F-value* of 7.5 and a *Prob > F* of 0.01. At the agreed significance level, these results indicate statistically significant differences between the groups in means (pairs). Thus, the cooling conditions exert a statistically significant influence on the mass of the processed material.

For processed mass parameter, Fisher’s test (Table A4, Appendix A) identified statistically significant differences (*Sig* = 1) for the following pairwise comparisons: WET vs. REF (*MeanDiff* = 13.33, *Prob* = 0.003), MQL vs. REF (*MeanDiff* = 8.67, *Prob* = 0.026), and HYB vs. REF (*MeanDiff* = 13, *Prob* = 0.004). Similarly to the knife operating time results, all examined cooling conditions (WET, MQL, HYB) differ significantly from the reference condition with respect to their impact on the mass of processed raw material. These findings are consistent with the visual representation in the Fisher LSD plot (Figure 9b).

The next stage was the ANOVA analysis of the plaice (*Pleuronectes platessa*), starting with the operating time of the knife in the skinning process. Although the *F-value* of 2.3 and *Prob > F* of 0.154 (Table 10) indicate no statistically significant differences among the mean knife operating times across different cooling conditions, Fisher’s test (Table A2, Appendix A) merits closer examination. Fisher’s test revealed statistically significant differences (*Sig* = 1) for the comparisons WET vs. REF (*MeanDiff* = 14, *Prob* = 0.084) and MQL vs. WET (*MeanDiff* = −15.67, *Prob* = 0.058).

In this case, an elevated significance level (*α* = 0.1) was adopted to increase the sensitivity of the tests to potential differences, taking into account the specific characteristics of the skinning process for this fish species and the observed data variability. A higher significance level allows the detection of trends that might be overlooked under a more stringent confidence threshold.

**Figure 10:**
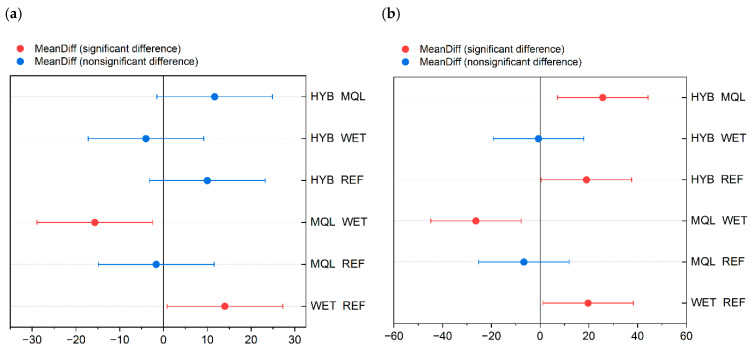
Means comparison plot for four different cooling strategies: reference (REF), conventional flood cooling (WET), minimum quantity lubrication (MQL), and hybrid cooling (HYB), with respect to: (**a**) the lifetime of technical knives, and (**b**) the mass of processed plaice fish (*Pleuronectes platessa*).

These results suggest that the WET and MQL cooling conditions significantly affect knife operating time relative to the reference condition and mutually in comparison, which is also visually confirmed by the Fisher LSD plot (Figure 10a), where the *MeanDiff* points for WET vs. REF and MQL vs. WET are marked as significant differences. Other comparisons did not show significant differences.

The final output factor analyzed for plaice is the mass of processed raw material during skinning. The overall ANOVA (Table 10) yielded an *F-value* of 3.59 and *Prob>F* of 0.065, indicating statistically significant differences in population means at the significance level of *α* = 0.1. This signifies that cooling conditions have a statistically significant impact on the mass of processed material.

Analysis of Fisher’s test (Table A2, Appendix A) revealed statistically significant differences (*Sig* = 1) for the comparisons WET vs. REF (*MeanDiff* = 19.67, *Prob* = 0.083), MQL vs. WET (*MeanDiff* = −26.33, *Prob* = 0.029), HYB vs. REF (*MeanDiff* = 19, *Prob* = 0.093), and HYB vs. MQL (*MeanDiff* = 25.67, *Prob* = 0.032). These results strongly indicate that the cooling conditions WET, MQL, and HYB significantly influence the mass of processed raw material, both in comparison to the reference condition and among themselves. The Fisher LSD plot (Figure 10b) also clearly illustrates these significant differences.

### 3.2. Comparative Assessment of Tool Life and Material Removal Under Various Cooling Strategies

The conducted research demonstrated that the application of appropriately selected cooling and lubrication conditions during the grinding process of planar knives results in a significant increase in tool life compared to commercial (reference) knives. In the plaice (*Pleuronectes platessa*) case, the average extension of tool life amounted to **+12.39% for the conventional flood cooling method (WET)** and **+8.85% for the hybrid method (MQL + CAG)**.

These findings indicate that the grinding parameters for edge shaping of planar technical knives, established through preliminary and principal investigations, enable a noticeable improvement in tool life for these two cooling and lubrication strategies when applied in the processing zone during the flatfish skinning operation.

This effect can be directly attributed to the influence of cooling conditions on the course of physical and mechanical phenomena in the grinding zone, particularly with regard to mitigating adverse thermomechanical effects. The WET method provides intensive cooling of the entire grinding area, effectively dissipating heat and reducing the risk of thermal overload. This leads to lower temperature gradients and the formation of less detrimental residual stresses. As a result, this method proved to be the most effective, yielding the longest average tool life—127 min, which is 8.5 min longer than the overall average.

The application of the MQL method significantly reduces the consumption of cooling–lubricating fluid (1100 mL per hour) compared to the flood cooling method (1750 mL per minute), which substantially limits the cooling function in the processing zone for this technique. Although MQL effectively reduces friction, its capacity to dissipate heat from the grinding zone is insufficient relative to the flood cooling method, where large volumes of coolant flush and remove heat along with grinding debris. Consequently, local overheating of the surface layer may occur during MQL processing, even if such overheating is not directly visible as burn marks. Any defects on the cutting-edge surface of the knives may remain undetected by conventional roughness or hardness measurements. However, this can weaken the tool life during actual contact with the processed material—in this case, fish skin and possible contaminants.

Due to elevated temperatures and insufficient flushing of grinding debris, the MQL method may also lead to distortions in the microgeometry of the cutting edges—such as microdeformations, including burrs and scratches caused by mechanical interaction with debris—which are not visible at the macroscopic scale but can negatively affect cutting performance [24]. In the flatfish skinning process, where knives must maintain exceptionally high sharpness and surface smoothness, even minimal degradation of the micro-edges can lead to a reduction in tool service life.

Despite the absence of an absolute extension in tool life for knives regenerated under minimum quantity lubrication (MQL) conditions compared to new (commercial) knives, the research findings unequivocally confirm the high effectiveness of the applied regeneration technology. Maintaining tool life comparable to that of new (reference) tools provides clear evidence of the appropriate selection of technological parameters in the grinding process. This demonstrates the preservation of the proper quality of the knife’s surface layer and cutting edge’s sharpness, consistent with the original functional requirements of commercial knives. Consequently, the application of the MQL method can be justified on ecological grounds: the elimination of large volumes of coolant reduces the burden on filtration and disposal systems, resulting in lower environmental costs; furthermore, effective knife regeneration enables multiple refurbishments and extended usage, thereby reducing the demand for manufacturing new tools.

The operational test results further suggest that supplementing the MQL method with additional cooling—through the supply of chilled compressed air generated by the CAG nozzle—effectively enhances heat dissipation from the machining zone, enabling the grinding of technical knife edges with a quality nearly comparable to that achieved under flood cooling (WET) conditions. The obtained increase in tool life averaged 10 min (+8.85%), alongside an average of 12.02% more processed raw material (equivalent to 11 kg more than the overall average). It should be emphasized that the consumption of liquid cooling–lubricating agent in this case is reduced by more than 95 times (from 105 L per hour to 1.1 L per hour), with simultaneous usage of approximately 3000 L of chilled compressed air per hour. Unlike liquid cooling lubricants, the use of air as a cooling medium does not incur costs related to purchase or disposal.

It can be inferred that the hybrid method (HYB) combines the lubricating effect of an oil aerosol (friction reduction) with the cooling effect of adiabatically expanded air, which effectively lowers the temperature in a more targeted and localized manner. The limited local overheating of the material due to precise cooling and efficient lubrication reduces the intensity of surface defects such as burr formation and microcracking, as well as the risk of formation of tempered zones. This results in a smoother working surface of the knife edges, which consequently translates into lower cutting resistance and slower wear under actual operating conditions.

Table A3 in Appendix A provides a summary of the operational test results for planar knives employed in the skinning of flounder (*Platichthys flesus*). The obtained data confirm the potential for extending tool life through precise edge shaping. However, the absolute values recorded for tool life in the processing of flounder remain significantly lower compared to those achieved during the skinning of plaice (*Pleuronectes platessa*).

In total, all tested tools operated for a cumulative duration of 604 min, enabling the processing of 1005 kg of fish raw material. The average operating time per individual blade was 50.33 min, while the mean mass of processed material amounted to 83.75 kg. By comparison, previous studies concerning the skinning of plaice (*Pleuronectes platessa*) yielded approximately twice the values for both parameters, which confirms the significant influence of fish species on blade wear.

The muscle tissue of flounder is characterized by a dense and more fibrous structure compared to the delicate musculature of plaice. The presence of collagen within the flounder’s muscle fibers increases their resistance to cutting, thereby necessitating higher cutting forces and intensifying wear-related phenomena. This structural resistance complicates the skin removal process and generates elevated mechanical loads acting on the cutting edge. An additional factor negatively affecting tool life was the above-average contamination of the removed skin, which likely accelerated edge dulling. Under such conditions, the cutting edge must perform increased mechanical work, resulting in more rapid edge degradation. Consequently, the tool life of both reference and refurbished knives was significantly reduced.

Despite these challenges, the obtained results confirm the effectiveness of tool refurbishment through appropriate edge geometry shaping. The study demonstrated the potential to extend the average operating time of knives in the flounder skinning process by 13.3% when applying grinding with Minimum Quantity Lubrication (MQL) cooling, and by 17.7% in the case of conventional flood (WET) cooling grinding.

The study results indicated not only an increase in operating time but also an improvement in material yield. The average mass of processed fish raw material increased when using knives labeled as WET, MQL, and HYB (ground using the MQL+CAG hybrid method), by 17.8%, 11.5%, and 17.3%, respectively (Figure 8b).

It is noteworthy that the relative increases in tool life and operational efficiency obtained for the flounder skinning process exceed those recorded for plaice. In the case of the WET method, the improvement amounts to approximately +5 percentage points in favor of the flounder results (17.7% compared to 12.39%). For the hybrid cooling method applied during grinding, the percentage increase in knife operating time until loss of cutting ability was nearly two-fold (+16.3% compared to +8.85%). Particularly significant is the result achieved with the grinding process utilizing Minimum Quantity Lubrication (MQL) cooling. In this case, the flounder skinning process yielded an increase in tool life (with operating time extended by approximately 13% and processed material yield reaching 11.5%) compared to new commercial knives. This makes the MQL cooling method both economically and technologically advantageous.

Extending the tool life of cutting blades provides measurable benefits to fish processing enterprises, primarily by reducing the number of knives consumed, which in turn leads to lower procurement costs and decreased frequency of production downtimes associated with blade replacement.

Based on operational data from Espersen Poland Ltd., Table 11 presents the average annual number of knives used in the production process during the years 2023–2024. Currently, this figure amounts to approximately 4240 units per year, corresponding to 282.67 h dedicated to blade replacement. This time estimate is based on the observation that each knife replacement takes an average of 4 min, as determined through direct time measurements conducted during operational testing under industrial conditions.

The time losses associated with tool changeovers are therefore substantial and can negatively impact the continuity of the technological process. Enhancing blade durability can thus generate both material savings (fewer knives consumed) and time savings (reduced downtime), which may directly contribute to improved production efficiency and reduced operational costs.

The data indicate that increasing the tool life of knives enables a reduction in their consumption by 508 units in the plaice (*Pleuronectes platessa*) skinning process and by 720 units in the case of European flounder (*Platichthys flesus*). This corresponds to time savings of 2035 min (approximately 34 working hours) and 2883 min (approximately 48 working hours) per year, respectively. These saved hours can be reallocated to enhance production line efficiency or to perform other tasks assigned to operators.

A greater potential gain was observed in the flounder processing operation—likely due to the higher rate of knife wear during its processing, which means that even a modest improvement in tool life yields significant benefits. The results unequivocally indicate that investing in more durable knives through the optimization of sharpening processes can deliver tangible economic and operational advantages to the company. Reducing both downtime and tooling costs represents a realistic opportunity to enhance the competitiveness of the enterprise.

## 4. Conclusions

The studies demonstrated that the durability of production cycles is influenced both by the species of the skinned fish fillet and by the cooling conditions applied during the cutting edge’s reconditioning. Due to the specific characteristics of the raw material, skinning flounder (*Platichthys flesus*) proved to be more demanding, resulting in approximately half the amount of processed material compared to the skinning of plaice (*Pleuronectes platessa*). Despite this difference, the developed methodology demonstrated superiority over the recommended solution (replacement of knives with new, commercially manufactured and sharpened tools).

Only in the case of applying Minimum Quantity Lubrication (MQL) cooling can it be expected that the reconditioning process will not yield the anticipated extension of the tool life of technical knives or will be limited to an increase of 13.5%, as observed in the flounder skinning process. The recommended cooling method during grinding remains flood cooling (WET), which provides the best results.

Selected detailed conclusions are presented as follows:

- In the case of edge shaping of knives during grinding performed under conditions established based on previously conducted research results, it can be concluded that the cutting properties of the blades are correctly restored, at least to the level characteristic of the reference knives. This enables the reuse of knives in the flatfish skinning process while simultaneously ensuring an extended operational cycle for these tools in most cases.
- Operational testing of technical knives, regardless of the cooling method applied during their edge shaping process, demonstrated very good cutting performance of the blades. The possibility of multiple reuses of the same knives translates into tangible economic and operational benefits, such as a reduction in the number of tool changeovers.
- Edge shaping of planar blades of technical knives under flood cooling (WET) and hybrid cooling methods (combining MQL and CAG) allowed an extension of the tool life during the skinning of plaice (*Pleuronectes platessa*) by 12.39% and 8.85%, respectively. This gain is significant enough to correspond both to a comparable increase in the quantity of processed raw material and to time savings of approximately 2035 min per year (about 34 working hours), resulting from the reduced frequency of production stoppages for tool replacement. The relative increase in effective tool life of refurbished technical knives in the flounder skinning process (*Platichthys flesus*) is even greater, amounting to 17.7% and 16.3% for the WET and HYB methods, respectively. The assumed annual time savings for changeovers in this case reach as much as 2883 min, approximately 48 working hours.
- Supplementing the MQL cooling method during grinding with additional cooling by supplying chilled compressed air generated through the CAG nozzle effectively improves heat dissipation from the machining zone. This allows for the formation of technical blades whose operational tool life is only slightly lower than that of knives shaped under flood cooling (WET) conditions. This difference ranges from −3.7% to −12% in tool life in favor of the flood cooling method. Importantly, the use of a hybrid cooling and lubrication system in the grinding zone enables over a 95-fold reduction in coolant consumption, significantly reducing costs associated with its purchase and disposal.
- The use of refurbished knives had no negative impact on the production process. It was observed that the processed fish fillets did not exhibit damage signs such as delamination. Organoleptic evaluation by experienced production personnel revealed no qualitative differences compared to the process conducted using reference knives.

Despite the favorable results obtained from operational studies conducted under industrial conditions, previously identified needs for further improvement in the planar knife sharpening process remain relevant to enable its continued optimization. It is recommended to extend the scope of research to include other fish species as well as a broader range of grinding conditions, which would allow for the development of models encompassing input factor values beyond the current experimental range.

Additionally, consideration should be given to the application of alternative multi-criteria optimization techniques. For example, a comparative analysis of the effectiveness of the MO-Jaya algorithm against genetic algorithms, or the utilization of recent advancements such as the memory-guided Jaya algorithm or the Aspiration Level-based Multi-Objective Quasi-Oppositional Jaya algorithm, may prove beneficial. The latter method enables incorporation of user preferences regarding desired objective function values, thereby directing the optimization process toward solutions that fulfill specified user requirements.

## Figures and Tables

**Table 1:** Physical and storage characteristics of *Pleuronectes platessa* and *Platichthys flesus* specimens used in industrial skinning experiments.

Characteristic	Plaice (*Pleuronectes platessa*)	Flounder (*Platichthys flesus*)
Weight before filleting (whole fish)	218 to 283 g	242 to 305 g
Weight before skinning (single fillet)	90 to 113 g	44 to 51 g
Storage form	Delivered fresh (not frozen during transport)	Delivered frozen (subjected to thawing at the processing plant)
Temperature during transport	0–2 °C	−18 °C

**Table 2:** Chemical composition of martensitic stainless steel X39Cr13 (1.4031) according to BS EN 10088-3:2023 [30].

Cr	C	Si	P	S	Mn
12.5 to 14.5	0.36 to 0.42	max. 1.00	max. 0.04	max. 0.03	max. 1.00

**Table 3:** Physical and mechanical properties of martensitic stainless steel X39Cr13 (1.4031) according to BS EN 10088-1:2014 [31] and BS EN 10088-3:2023 [30].

Density at 20 °C	Modulus of elasticity at 20 °C	Heat conductivity coefficient	Specific heat at 20 °C	Electrical resistivity at 20 °C
7.7 kg/dm^3^	215 GPa	30 W/m·K	460 J/kg·K	0.55 (Ω·mm^2^)/m
Brinell hardness (HB)	Yield strength (0.2% offset, *Rp_0.2_*)	Tensile strength (*R_m_*)	Elongation after fracture (*A*)	Impact energy (*KV*) (ISO-V, longitudinal)
max. 245	650 MPa	800 to 1000 MPa	min. 10%	*KV* ≥ 12 J

**Table 4:** Set of selected Pareto-optimal solutions (for MOJaya parameters: populationSize = 10, dimensions = 2, and maxGenerations = 20).

*v_f_* (mm/min)	*a_es_* (mm)	*P* (W)	*F_c_* (N)	||A||_F_
100	0.03	49.10	11.91	29.63
100	0.03	49.10	11.91	29.63
100	0.02	19.46	12.30	0.39
100	0.02	19.46	12.30	0.39
100	0.03	49.10	11.91	29.63
100	0.02	19.46	12.30	0.39
100	0.03	49.10	11.91	29.63
100	0.03	49.10	11.91	29.63
100	0.02	19.46	12.30	0.39

**Table 5:** Constant parameters of the peripheral grinding process for planar knives.

Grinding Wheel Rotational Speed (*n_s_*)	Allowance for Rough Pass (*a_e_*)	Allowance for Spark-Out Pass (*a_es_*)	Grinding Wheel Longitudinal Feed Rate (*v_f_*)
32,000 rev/min	0.1 mm	0.02 mm	100 mm/min

**Table 6:** Designation of planar knife groups used in operational tests and their corresponding cooling conditions in the grinding process.

Group	Label	Grinding Conditions	Lubricating Agent	Cooling Agent	Heat Capacity * (*C_p_*)
Reference knifes	RK	unknown	unknown	unknown	—
Ground knifes	WET	Flood cooling method	Fluid: 5% water solution of oil Oil: Emulgol ES-12 oil Flow rate (Orlen, Plock, Poland): 1750 mL per min (105 L per hour)		Water: 4.18 kJ/kg·K
	MQL	Minimum quantity lubrication with coolant–lubricant fluid using an MQL-type Micro-Jet MKS-G100 system manufactured by MicroJet (Karlsruhe, Germany)	Oil-mist Oil: Cimtech (Cimcool Industrial Products, Vlaardingen, The Netherlands)^®^ MQL Supply air pressure: 0.6 MPa Flow rate: 1100 mL per hour		Oil: 1.92 kJ/kg·K
	HYB	Minimum quantity lubrication with coolant–lubricant fluid using an MQL nozzle combined with cooling by cooled compressed air supplied through a CAG nozzle	Emulsion (Water+Oil) Oil: Cimtech^®^ MQL Supply air pressure: 0.6 MPa Flow rate: 1100 mL per hour	Compressed cooled air Nozzle: Vortec 610 Temperature of CCA: −5 °C Flow rate: 50 L per minute	Oil: 1.92 kJ/kg·K Air: 1.04 kJ/kg·K

* Heat capacity according to [35].

**Table 7:** Skinning process parameters and environmental conditions.

Parameter	Value
Ambient temperature	16–18 °C
Temperature of running water (rinsing nozzles of the skinning machine and manual filleting table)	2–5 °C
Feed conveyor speed	10 m/min
Skinning machine conveyor speed	19 m/min
Flow rate of rinsing water from skinning machine nozzles	2.5 dm^3^/min

**Table 8:** Designations of planar knife groups selected for operational testing and their corresponding grinding cooling conditions.

Group	Labels	Grinding Conditions *
Reference knifes	REF#1 to REF#6	unknown
Ground knifes	WET#1 to WET#6	Flood cooling method (WET)
	MQL#1 to MQL#6	Minimum quantity lubrication (MQL) cooling-lubrication using an MQL nozzle
	HYB#1 to HYB#6	Minimum quantity lubrication (MQL) cooling-lubrication combined with cooled compressed air cooling using a CAG nozzle

* More details are provided in Section 2.3.

**Table 9:** Overall ANOVA analysis results for different cooling conditions in the case of flounder (*Platichthys flesus*).

	Degrees of Freedom	Sum of Squares	Mean Square	*F*-Value	*Prob > F* *
*Lifetime of the technical knife*					
Model	3	120	40	7.16	0.012
Error	8	44.67	5.58		
Total	11	164.67			
*Processed mass of fish*					
Model	3	346.92	115.64	7.50	0.010
Error	8	123.33	15.42		
Total	11	470.25			

* *Prob* > *F* represents the probability that the obtained result is due to chance (*p*-value for F-test).

**Table 10:** Overall ANOVA analysis results for different cooling conditions in the case of plaice (*Pleuronectes platessa*).

	Degrees of Freedom	Sum of Squares	Mean Square	*F*-Value	*Prob > F* *
*Lifetime of Technical Knife*					
Model	3	522.25	174.08	2.30	0.154
Error	8	604.67	75.58		
Total	11	1126.92			
*Processed mass of fish*					
Model	3	1608.67	536.22	3.59	0.065
Error	8	1193.33	149.17		
Total	11	2802			

* *Prob > F* represents the probability that the obtained result is due to chance (*p*-value for F-test).

**Table 11:** Summary of the number of knives consumed and average replacement times (data from Espersen Poland Ltd. for the years 2023–2024).

Parameter	Value	
Average annual number of knives consumed	4240 pieces per year	
Total tool replacement time (approx. 4 min per piece)	282.67 h per year	
Raw material species	Plaice (*Pleuronectes platessa*)	Flounder (*Platichthys Flesus*)
Percentage gain (in case of extended tool life)	+12%	+17%
Estimated average reduction in the number of knives consumed (in case of extended tool life)	508 pieces per year	720 pieces per year
Estimated average production time saved (in case of extended tool life)	2035 min per year	2883 min per year

## Data Availability

The data presented in this study are available on request from the corresponding author because they require licensed software to process them.

## Nomenclature

The following abbreviations and symbols are used in this manuscript:

CAG	grinding fluid in the form of cooled air using cold air guns (cold compressed air method)
HSB	color space; HSB stands for hue, saturation, and brightness
IQR	interquartile range (a measure of statistical dispersion)
LCL	lower confidence intervals of the parameter
MQL	technique of cooling in the grinding process with minimum quantity lubrication
UCL	upper confidence intervals of the parameter
SE	standard error of a statistic (estimator of a parameter)
WET	conventional flood method in the grinding process using water emulsion as coolant
α	statistical significance level
**||A||_F_**	distance from an ideal point (Frobenius distance)
*a**_e_*	machining allowance (working engagement) for rough passage, mm
*a**_es_*	allowance for sparking-out phase, mm
*F**_c_*	cutting force, N
*F*-value	result of a statistical test in ANOVA, where the test statistic follows Snedecor’s F-distribution
GT/GT*_val_*	grinding type (categorial variable); numerical value of category is GT*_val_*
*MeanDiff*	mean difference between data groups
*n**_s_*	grinding wheel rotational speed, rev/min
*P*	grinding power, W
*Prob*	probability of observing a test statistic
*Sig*	significance flag (indicates that a pair of groups is significantly different in a pairwise comparison test)
*t-value*	value of a statistical test obtained using Student’s *t*-test
*v**_f_*	feed speed in the grinding process; precisely the longitudinal feed speed of the grinding wheel, mm/min

## Appendix A

The following appendix contains experimental results that complement the analysis presented in Section 3, Results and Discussion. These data provide additional context and support for the conclusions drawn in the main text, offering detailed measurements that were omitted from the main body for clarity and conciseness. In particular, it includes full datasets, extended measurement series, and replicate results that underpin the representative figures discussed in the main text.

**Table A1:** Summary of knife operating time results in the skinning process of plaice (*Pleuronectes platessa*).

Group	Label	Lifetime (minutes)	Avg. Time (minutes)	SE of Mean (minutes)	Processed Mass (kg)	Avg. Mass (kg)	SE of Mean (kg)
Reference knifes	REF#1	105	113	5.29	147	158	7.37
	REF#2	123			172		
	REF#3	111			155		
	*Sum:*	*339*	*—*	*—*	*474*	—	—
Ground knifes	WET#1	135	127	4.16	189	177.66 (6)	5.93
	WET#2	121			169		
	WET#3	125			175		
	*Sum:*	*381*	*—*	*—*	*533*	—	—
	MQL#1	114	111.33 (3)	4.81	155	151.33 (3)	6.33
	MQL#2	118			160		
	MQL#3	102			139		
	*Sum:*	*334*	*—*	*—*	*454*	—	—
	HYB#1	126	123	5.69	181	177	8.32
	HYB#2	112			161		
	HYB#3	131			189		
	*Sum:*	*369*	*—*	*—*	*531*	—	—
Overall:	—	1423	118.58	2.92	1992	166	4.61

**Table A2:** Fisher’s LSD test results for plaice (*Pleuronectes platessa*).

Groups of Means (pairs)	*MeanDiff* *	*t*-Value	*Prob* **	*Sig* ***	LCL	UCL
	*Lifetime of Technical Knife*					
WET-REF	14	1.97	0.084	1	0.79	27.20
MQL-REF	−1.67	−0.23	0.820	0	−14.86	11.53
MQL-WET	−15.67	−2.20	0.058	1	−28.86	−2.46
HYB-REF	10	1.41	0.196	0	−3.20	23.20
HYB-WET	−4	−0.56	0.588	0	−17.20	9.20
HYB-MQL	11.67	1.64	0.138	0	−1.53	24.86
	*Processed mass of fish*					
WET-REF	19.67	1.97	0.083	1	1.12	38.21
MQL-REF	−6.67	−0.67	0.523	0	−25.21	11.87
MQL-WET	−26.33	−2.64	0.029	1	−44.87	−7.78
HYB-REF	19	1.90	0.093	1	0.45	37.54
HYB-WET	−0.67	−0.07	0.948	0	−19.21	17.87
HYB-MQL	25.67	2.57	0.032	1	7.12	44.21

* *MeanDiff*—mean difference between data groups. ** *Prob*—probability of observing a test statistic. *** *Sig*—significance.

**Table A3:** Summary of knife operating time results in the skinning process of flounder (*Platichthys flesus*).

Group	Label	Lifetime (minutes)	Avg. Time (minutes)	SE of Mean (minutes)	Processed Mass (kg)	Avg. Mass (kg)	SE of Mean (kg)
Reference knifes	REF#4	48	45	1.53	80	75	2.52
	REF#5	44			73		
	REF#6	43			72		
	*Sum:*	*135*	*—*	*—*		—	—
Ground knifes	WET#4	51	53	1.53	85	88.33 (3)	2.40
	WET#5	56			93		
	WET#6	52			87		
	*Sum:*	*159*	*—*	*—*		—	—
	MQL#4	53	51	1.15	87	83.66 (6)	2.03
	MQL#5	51			84		
	MQL#6	49			80		
	*Sum:*	*153*	*—*	*—*		—	—
	HYB#4	50	52.33 (3)	1.20	84	88	2.08
	HYB#5	54			91		
	HYB#6	53			89		
	*Sum:*	*157*	*—*	*—*		—	—
Overall:	—	604	50.33 (3)	1.12	1005	83.75	1.89

**Table A4:** Fisher’s LSD test results for flounder (*Platichthys flesus*).

Groups of Means (pairs)	*MeanDiff* *	*t*-Value	*Prob* **	*Sig* ***	LCL	UCL
	*Lifetime of Technical Knife*					
WET-REF	8	4.14	0.003	1	3.55	12.45
MQL-REF	6	3.11	0.014	1	1.55	10.44
MQL-WET	−2	−1.03	0.330	0	−6.45	2.45
HYB-REF	7.33	3.80	0.005	1	2.88	11.78
HYB-WET	−0.67	−0.34	0.738	0	−5.12	3.78
HYB-MQL	1.33	0.69	0.509	0	−3.11	5.78
	*Processed mass of fish*					
WET-REF	13.33	4.16	0.003	1	5.94	20.72
MQL-REF	8.67	2.70	0.026	1	1.27	16.05
MQL-WET	−4.67	−1.45	0.183	0	−12.06	2.72
HYB-REF	13	4.05	0.004	1	5.61	20.39
HYB-WET	−0.33	−0.10	0.919	0	−7.72	7.05
HYB-MQL	4.33	1.35	0.213	0	−3.05	11.72

* *MeanDiff*—mean difference between data groups. ** *Prob*—probability of observing a test statistic. *** *Sig*—significance.
